# 

**Published:** 1899-08-26

**Authors:** 


					THB HOSPITAL. " The standard of highest purity."?Lancet. August 26th, 1899
CADBURY'S pif V jp OADBURY'S
COGOA 9k ^ ^ COCOA
A?SOiSt!lT FORK. ^ NO DRUGS OR ALKALIES OSED.
No. 6j4. Vol. XXVI. [fCpfp0n] Saturday,
August 26th, 1899,
j
L^scnp^nwj pBICE TWOpEIfCE>

				

